# *N*-*tert*-Butyl-5α-androstane-17β-carboxamide

**DOI:** 10.1107/S1600536809005741

**Published:** 2009-02-25

**Authors:** Xin Yan, Shiqing Xu, Jingmei Wang, Ying Chen, Peng Xia

**Affiliations:** a Department of Medicinal Chemistry, School of Pharmacy, Fudan University, Shanghai 200032, People’s Republic of China; b Center of Analysis and Measurement, Fudan University, Shanghai 200433, People’s Republic of China

## Abstract

The title compound, C_24_H_41_NO, is a new derivative of the anti-HIV steroid 17β-(*N*-*tert*-butyl­amino­carbon­yl)androst-4-en-3-one. There are four rings in the structure and these are *trans*-fused. The three six-membered rings exhibit chair conformations, while the five-membered ring adopts an envelope conformation.

## Related literature

For the anti-HIV activity of 17β-(*N*-*tert*-butyl­amino­carbon­yl)-androst-4-en-3-one, see: Xia *et al.* (1999 ▶). For discussion of absolute configuration, see: Marker *et al.* (1940 ▶); Fieser & Fieser (1959 ▶); Throop & Tokes (1967 ▶); House (1972 ▶); Castro-Méndez *et al.* (2002 ▶).
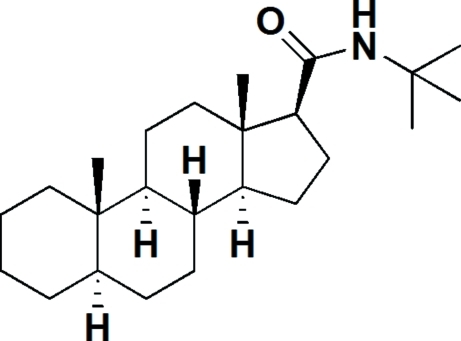


## Experimental

### 

#### Crystal data

C_24_H_41_NO
*M* *_r_* = 359.58
Orthorhombic, 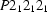

*a* = 6.373 (2) Å
*b* = 12.802 (4) Å
*c* = 26.775 (9) Å
*V* = 2184.3 (12) Å^3^
*Z* = 4
Mo *K*α radiation
μ = 0.07 mm^−1^
*T* = 294 K
0.15 × 0.08 × 0.06 mm

#### Data collection

Bruker SMART CCD area-detector diffractometer
Absorption correction: multi-scan (*SADABS*; Sheldrick, 1996 ▶) *T*_min_ = 0.990, *T*_max_ = 0.996
10583 measured reflections
2757 independent reflections
1433 reflections with *I* > 2σ(*I*)
*R*_int_ = 0.063

#### Refinement

*R*[*F* ^2^ > 2σ(*F* ^2^)] = 0.037
*wR*(*F* ^2^) = 0.068
*S* = 0.75
2757 reflections
240 parameters
H-atom parameters constrained
Δρ_max_ = 0.10 e Å^−3^
Δρ_min_ = −0.12 e Å^−3^

### 

Data collection: *SMART* (Bruker, 2000 ▶); cell refinement: *SAINT* (Bruker, 2000 ▶); data reduction: *SAINT*; program(s) used to solve structure: *SHELXS97* (Sheldrick, 2008 ▶); program(s) used to refine structure: *SHELXL97* (Sheldrick, 2008 ▶); molecular graphics: *SHELXTL* (Sheldrick, 2008 ▶); software used to prepare material for publication: *SHELXTL*.

## Supplementary Material

Crystal structure: contains datablocks I, global. DOI: 10.1107/S1600536809005741/tk2372sup1.cif

Structure factors: contains datablocks I. DOI: 10.1107/S1600536809005741/tk2372Isup2.hkl

Additional supplementary materials:  crystallographic information; 3D view; checkCIF report

## Figures and Tables

**Fig. 1.**
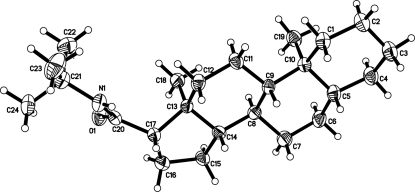
The molecular structure of (I), with atom labels and 30% probability displacement ellipsoids for non-H atoms.

## supplementary crystallographic information

### Comment

17β-(*N*-*tert*-Butylcarboxamide)-androst-4-ene-3-one was reported to exhibit potent anti-HIV activity in acutely infected H_9_ lymphocytes with EC_50_ and therapeutic index values of 0.8 and 300 *µ*M, respectively (Xia *et al.*,  1999). During our work of structural modification, which is motivated by the search for more  potent anti-HIV agents, we found that 17β-(*N*-*tert*-butylcarboxamide)-5α-androstane (I) could be obtained through Pd/C catalytic hydrogenation of 17β-(*N*-*tert*-butylcarboxamide)-androst-4-ene-3-ol in excellent yield (90%); full structural details of (I) are reported herein.

The molecular structure of (I), Fig.1, shows the A/B, B/C and C/D ring junctions to be all trans. The cyclohexane rings adopt chair conformations, and the cyclopentane ring adopts an envelope conformation. Based on the known configurations of the C10, C13-methyl groups, see Experimental, 5-H is assigned an α-configuration. The 17-*N*-*tert*-butylcarboxamide group is in a β-configuration. The stereogenic sites of (I) exhibit the following chirality: C5 = R, C8 = R, C9 = S, C10 = S, C13 = S, C14 = S and C17 = S.

### Experimental

Compound (I) was prepared from the corresponding 4-ene-3-ol by catalytic hydrogenation with 5% palladium-on-charcoal in EtOH for 1 day. After filtration and removal of the solvent, the residue was crystallized from acetone to give colourless crystals.

The starting material, 17β-(*N*-*tert*-butylcarboxamide)-androst-4-ene-3-ol, was obtained from the reduction of 17β-(*N*-*tert*-butylcarboxamide)-androst-4-ene-3-one with NaBH_4_. It is an intermediate in the synthesis of Finasteride and derived initially from diosgenin, for which the absolute configurations of all chiral centers of the steroid skeleton have been determined (Fieser & Fieser, 1959; Marker *et al.*, 1940). Recently, the absolute configurations of the chiral centres were confirmed by the X-ray crystal structure determination of a 3-Br substituted steroid substrate synthesized from diosgenin (Castro-Méndez *et al.*, 2002). The hydrogenation of 4-en-3-one moiety did not cause inversion of the configurations at C8, C9, C10, C13 and C14 (Throop & Tokes, 1967; House, 1972). Thus, by comparing the orientation of 5-H to that of methyl groups at C10 and C13, the absolute configuration of (I) could be determined.

### Refinement

All H atoms were placed in the idealized positions with N—H = 0.86 Å, methine C—H = 0.98 Å, methylene C—H = 0.97 Å and methyl C—H = 0.96 Å, and treated as riding with *U*_iso_(H) = 1.2 *U*_eq_(N-H, CH_2_ and CH) and 1.5*U*_eq_(CH_3_). In the absence of significant anomalous scattering effects, 1971 Friedel pairs were averaged in the final refinement.

### Crystal data

**Table:**

C_24_H_41_NO	*D*_x_ = 1.093 Mg m^−^^3^
*M**_r_* = 359.58	Melting point: 451.5 K
Orthorhombic, *P*2_1_2_1_2_1_	Mo *K*α radiation, λ = 0.71073 Å
Hall symbol: P 2ac 2ab	Cell parameters from 962 reflections
*a* = 6.373 (2) Å	θ = 2.8–19.0°
*b* = 12.802 (4) Å	µ = 0.07 mm^−^^1^
*c* = 26.775 (9) Å	*T* = 294 K
*V* = 2184.3 (12) Å^3^	Parallelepiped, colourless
*Z* = 4	0.15 × 0.08 × 0.06 mm
*F*(000) = 800	

### Data collection

**Table:**

Bruker SMART CCD area-detector diffractometer	2757 independent reflections
Radiation source: sealed tube	1433 reflections with *I* > 2σ(*I*)
graphite	*R*_int_ = 0.063
φ and ω scans	θ_max_ = 27.1°, θ_min_ = 1.5°
Absorption correction: multi-scan (*SADABS*; Sheldrick, 1996)	*h* = −8→8
*T*_min_ = 0.990, *T*_max_ = 0.996	*k* = −16→16
10583 measured reflections	*l* = −34→22

### Refinement

**Table:**

Refinement on *F*^2^	Primary atom site location: structure-invariant direct methods
Least-squares matrix: full	Secondary atom site location: difference Fourier map
*R*[*F*^2^ > 2σ(*F*^2^)] = 0.037	Hydrogen site location: inferred from neighbouring sites
*wR*(*F*^2^) = 0.068	H-atom parameters constrained
*S* = 0.75	*w* = 1/[σ^2^(*F*_o_^2^) + (0.0299*P*)^2^] where *P* = (*F*_o_^2^ + 2*F*_c_^2^)/3
2757 reflections	(Δ/σ)_max_ < 0.001
240 parameters	Δρ_max_ = 0.10 e Å^−^^3^
0 restraints	Δρ_min_ = −0.12 e Å^−^^3^

### Special details

**Table:**

Geometry. All e.s.d.'s (except the e.s.d. in the dihedral angle between two l.s. planes) are estimated using the full covariance matrix. The cell e.s.d.'s are taken into account individually in the estimation of e.s.d.'s in distances, angles and torsion angles; correlations between e.s.d.'s in cell parameters are only used when they are defined by crystal symmetry. An approximate (isotropic) treatment of cell e.s.d.'s is used for estimating e.s.d.'s involving l.s. planes.
Refinement. Refinement of *F*^2^ against ALL reflections. The weighted *R*-factor *wR* and goodness of fit *S* are based on *F*^2^, conventional *R*-factors *R* are based on *F*, with *F* set to zero for negative *F*^2^. The threshold expression of *F*^2^ > σ(*F*^2^) is used only for calculating *R*-factors(gt) *etc*. and is not relevant to the choice of reflections for refinement. *R*-factors based on *F*^2^ are statistically about twice as large as those based on *F*, and *R*- factors based on ALL data will be even larger.

### Fractional atomic coordinates and isotropic or equivalent isotropic displacement parameters (Å^2^)

**Table:**

	*x*	*y*	*z*	*U*_iso_*/*U*_eq_	
N1	0.8035 (3)	0.65842 (13)	0.56653 (6)	0.0540 (5)	
H1	0.9247	0.6820	0.5751	0.065*	
O1	0.4554 (3)	0.67334 (13)	0.58044 (5)	0.0666 (5)	
C1	1.1936 (4)	0.62769 (17)	0.84810 (7)	0.0534 (6)	
H1A	1.2184	0.5667	0.8274	0.064*	
H1B	1.2916	0.6815	0.8377	0.064*	
C2	1.2381 (4)	0.59935 (18)	0.90230 (7)	0.0614 (7)	
H2A	1.1548	0.5388	0.9114	0.074*	
H2B	1.3849	0.5806	0.9057	0.074*	
C3	1.1892 (4)	0.6872 (2)	0.93761 (8)	0.0682 (7)	
H3A	1.2928	0.7420	0.9334	0.082*	
H3B	1.1993	0.6618	0.9717	0.082*	
C4	0.9727 (4)	0.73240 (19)	0.92929 (7)	0.0635 (7)	
H4A	0.9568	0.7949	0.9494	0.076*	
H4B	0.8681	0.6822	0.9402	0.076*	
C5	0.9344 (4)	0.75945 (17)	0.87460 (7)	0.0522 (6)	
H5	1.0430	0.8106	0.8660	0.063*	
C6	0.7298 (4)	0.81485 (17)	0.86688 (7)	0.0610 (7)	
H6A	0.7239	0.8752	0.8887	0.073*	
H6B	0.6157	0.7685	0.8760	0.073*	
C7	0.7000 (4)	0.85044 (16)	0.81299 (7)	0.0614 (7)	
H7A	0.5601	0.8792	0.8091	0.074*	
H7B	0.8002	0.9053	0.8055	0.074*	
C8	0.7295 (3)	0.76086 (15)	0.77615 (7)	0.0426 (5)	
H8	0.6196	0.7091	0.7826	0.051*	
C9	0.9436 (3)	0.70738 (15)	0.78447 (6)	0.0397 (5)	
H9	1.0496	0.7621	0.7802	0.048*	
C10	0.9693 (3)	0.66681 (15)	0.83881 (7)	0.0400 (5)	
C11	0.9900 (4)	0.62511 (16)	0.74440 (7)	0.0508 (6)	
H11A	0.9022	0.5646	0.7505	0.061*	
H11B	1.1350	0.6031	0.7477	0.061*	
C12	0.9537 (3)	0.66238 (17)	0.69070 (7)	0.0499 (6)	
H12A	1.0565	0.7154	0.6823	0.060*	
H12B	0.9722	0.6042	0.6679	0.060*	
C13	0.7344 (3)	0.70711 (14)	0.68449 (7)	0.0382 (5)	
C14	0.7092 (3)	0.79588 (15)	0.72231 (7)	0.0436 (5)	
H14	0.8250	0.8446	0.7162	0.052*	
C15	0.5092 (4)	0.85175 (17)	0.70618 (7)	0.0567 (6)	
H15A	0.5117	0.9245	0.7163	0.068*	
H15B	0.3863	0.8185	0.7205	0.068*	
C16	0.5093 (4)	0.84178 (16)	0.64878 (7)	0.0599 (7)	
H16A	0.5323	0.9095	0.6334	0.072*	
H16B	0.3760	0.8145	0.6372	0.072*	
C17	0.6889 (4)	0.76619 (14)	0.63516 (6)	0.0442 (6)	
H17	0.8130	0.8077	0.6266	0.053*	
C18	0.5691 (4)	0.62057 (15)	0.69106 (7)	0.0539 (6)	
H18A	0.5990	0.5642	0.6685	0.081*	
H18B	0.4323	0.6483	0.6840	0.081*	
H18C	0.5730	0.5952	0.7248	0.081*	
C19	0.8159 (4)	0.57727 (15)	0.84878 (8)	0.0584 (7)	
H19A	0.8546	0.5178	0.8290	0.088*	
H19B	0.6764	0.5989	0.8401	0.088*	
H19C	0.8204	0.5589	0.8835	0.088*	
C20	0.6366 (4)	0.69668 (16)	0.59120 (8)	0.0487 (6)	
C21	0.7988 (4)	0.58025 (17)	0.52642 (8)	0.0598 (7)	
C22	0.6959 (5)	0.48089 (17)	0.54580 (9)	0.0912 (9)	
H22A	0.7737	0.4550	0.5739	0.137*	
H22B	0.6947	0.4292	0.5198	0.137*	
H22C	0.5545	0.4959	0.5559	0.137*	
C23	1.0254 (5)	0.5606 (2)	0.51203 (10)	0.1023 (10)	
H23A	1.0853	0.6236	0.4988	0.153*	
H23B	1.0314	0.5066	0.4872	0.153*	
H23C	1.1031	0.5392	0.5410	0.153*	
C24	0.6785 (5)	0.6221 (2)	0.48153 (8)	0.0816 (8)	
H24A	0.5353	0.6348	0.4908	0.122*	
H24B	0.6828	0.5717	0.4550	0.122*	
H24C	0.7415	0.6861	0.4705	0.122*	

### Atomic displacement parameters (Å^2^)

**Table:**

	*U*^11^	*U*^22^	*U*^33^	*U*^12^	*U*^13^	*U*^23^
N1	0.0549 (14)	0.0628 (12)	0.0443 (11)	−0.0034 (11)	0.0022 (10)	−0.0105 (10)
O1	0.0572 (12)	0.0831 (11)	0.0595 (10)	−0.0006 (11)	−0.0099 (9)	−0.0130 (9)
C1	0.0520 (16)	0.0652 (14)	0.0430 (13)	0.0011 (13)	−0.0016 (12)	−0.0034 (11)
C2	0.0565 (18)	0.0790 (15)	0.0486 (14)	0.0058 (14)	−0.0050 (12)	0.0054 (13)
C3	0.075 (2)	0.0871 (18)	0.0425 (14)	0.0031 (17)	−0.0089 (14)	0.0036 (13)
C4	0.072 (2)	0.0781 (17)	0.0407 (14)	0.0077 (16)	0.0017 (13)	−0.0055 (12)
C5	0.0539 (17)	0.0644 (15)	0.0382 (13)	0.0022 (14)	0.0015 (12)	−0.0031 (11)
C6	0.069 (2)	0.0696 (15)	0.0443 (14)	0.0139 (14)	−0.0002 (12)	−0.0153 (12)
C7	0.0739 (18)	0.0619 (14)	0.0483 (14)	0.0217 (14)	0.0024 (13)	−0.0083 (12)
C8	0.0440 (15)	0.0451 (12)	0.0386 (12)	0.0060 (11)	0.0060 (10)	−0.0030 (10)
C9	0.0386 (13)	0.0454 (12)	0.0351 (12)	−0.0033 (11)	0.0036 (10)	−0.0027 (10)
C10	0.0367 (14)	0.0471 (12)	0.0363 (12)	−0.0011 (11)	0.0051 (10)	0.0010 (10)
C11	0.0466 (15)	0.0642 (14)	0.0415 (12)	0.0151 (12)	−0.0001 (11)	−0.0012 (11)
C12	0.0482 (15)	0.0620 (14)	0.0395 (12)	0.0051 (13)	0.0042 (11)	−0.0075 (11)
C13	0.0392 (14)	0.0419 (11)	0.0336 (11)	0.0017 (11)	0.0008 (10)	−0.0009 (10)
C14	0.0486 (15)	0.0413 (11)	0.0409 (12)	0.0021 (12)	0.0023 (11)	0.0032 (10)
C15	0.0699 (19)	0.0552 (14)	0.0451 (13)	0.0215 (14)	−0.0044 (12)	−0.0012 (11)
C16	0.0740 (19)	0.0529 (13)	0.0527 (14)	0.0134 (14)	−0.0027 (13)	0.0028 (12)
C17	0.0508 (15)	0.0464 (12)	0.0355 (12)	−0.0004 (12)	−0.0007 (11)	0.0017 (10)
C18	0.0607 (16)	0.0527 (13)	0.0485 (14)	−0.0046 (13)	0.0004 (12)	0.0035 (11)
C19	0.0584 (18)	0.0646 (15)	0.0522 (14)	−0.0089 (14)	−0.0024 (13)	0.0098 (12)
C20	0.0608 (18)	0.0484 (13)	0.0370 (13)	0.0006 (14)	0.0000 (13)	0.0067 (11)
C21	0.073 (2)	0.0573 (14)	0.0492 (14)	0.0029 (14)	0.0018 (13)	−0.0104 (13)
C22	0.135 (3)	0.0530 (15)	0.0855 (19)	−0.0078 (19)	0.0009 (19)	−0.0047 (14)
C23	0.085 (2)	0.124 (2)	0.098 (2)	0.017 (2)	0.016 (2)	−0.0481 (19)
C24	0.113 (2)	0.0870 (17)	0.0447 (14)	−0.0078 (18)	−0.0044 (16)	−0.0127 (14)

### Geometric parameters (Å, °)

**Table:**

N1—C20	1.345 (3)	C11—H11B	0.9700
N1—C21	1.468 (3)	C12—C13	1.519 (3)
N1—H1	0.8600	C12—H12A	0.9700
O1—C20	1.227 (3)	C12—H12B	0.9700
C1—C2	1.522 (3)	C13—C14	1.531 (3)
C1—C10	1.535 (3)	C13—C18	1.539 (3)
C1—H1A	0.9700	C13—C17	1.549 (3)
C1—H1B	0.9700	C14—C15	1.524 (3)
C2—C3	1.502 (3)	C14—H14	0.9800
C2—H2A	0.9700	C15—C16	1.542 (2)
C2—H2B	0.9700	C15—H15A	0.9700
C3—C4	1.513 (3)	C15—H15B	0.9700
C3—H3A	0.9700	C16—C17	1.542 (3)
C3—H3B	0.9700	C16—H16A	0.9700
C4—C5	1.524 (3)	C16—H16B	0.9700
C4—H4A	0.9700	C17—C20	1.513 (3)
C4—H4B	0.9700	C17—H17	0.9800
C5—C6	1.498 (3)	C18—H18A	0.9599
C5—C10	1.541 (3)	C18—H18B	0.9599
C5—H5	0.9800	C18—H18C	0.9599
C6—C7	1.525 (2)	C19—H19A	0.9599
C6—H6A	0.9700	C19—H19B	0.9599
C6—H6B	0.9700	C19—H19C	0.9599
C7—C8	1.524 (2)	C21—C23	1.516 (4)
C7—H7A	0.9700	C21—C24	1.523 (3)
C7—H7B	0.9700	C21—C22	1.522 (3)
C8—C14	1.515 (2)	C22—H22A	0.9599
C8—C9	1.542 (3)	C22—H22B	0.9599
C8—H8	0.9800	C22—H22C	0.9599
C9—C11	1.532 (2)	C23—H23A	0.9599
C9—C10	1.554 (2)	C23—H23B	0.9599
C9—H9	0.9800	C23—H23C	0.9599
C10—C19	1.530 (3)	C24—H24A	0.9599
C11—C12	1.532 (3)	C24—H24B	0.9599
C11—H11A	0.9700	C24—H24C	0.9599
			
C20—N1—C21	126.2 (2)	C13—C12—H12B	109.4
C20—N1—H1	116.9	C11—C12—H12B	109.4
C21—N1—H1	116.9	H12A—C12—H12B	108.0
C2—C1—C10	113.91 (17)	C12—C13—C14	107.69 (17)
C2—C1—H1A	108.8	C12—C13—C18	110.23 (17)
C10—C1—H1A	108.8	C14—C13—C18	112.79 (16)
C2—C1—H1B	108.8	C12—C13—C17	116.70 (16)
C10—C1—H1B	108.8	C14—C13—C17	100.48 (14)
H1A—C1—H1B	107.7	C18—C13—C17	108.70 (17)
C3—C2—C1	112.51 (19)	C8—C14—C15	118.68 (17)
C3—C2—H2A	109.1	C8—C14—C13	113.62 (16)
C1—C2—H2A	109.1	C15—C14—C13	104.39 (16)
C3—C2—H2B	109.1	C8—C14—H14	106.5
C1—C2—H2B	109.1	C15—C14—H14	106.5
H2A—C2—H2B	107.8	C13—C14—H14	106.5
C2—C3—C4	112.5 (2)	C14—C15—C16	104.07 (17)
C2—C3—H3A	109.1	C14—C15—H15A	110.9
C4—C3—H3A	109.1	C16—C15—H15A	110.9
C2—C3—H3B	109.1	C14—C15—H15B	110.9
C4—C3—H3B	109.1	C16—C15—H15B	110.9
H3A—C3—H3B	107.8	H15A—C15—H15B	109.0
C3—C4—C5	111.98 (19)	C15—C16—C17	106.73 (16)
C3—C4—H4A	109.2	C15—C16—H16A	110.4
C5—C4—H4A	109.2	C17—C16—H16A	110.4
C3—C4—H4B	109.2	C15—C16—H16B	110.4
C5—C4—H4B	109.2	C17—C16—H16B	110.4
H4A—C4—H4B	107.9	H16A—C16—H16B	108.6
C6—C5—C4	112.29 (18)	C20—C17—C16	112.92 (19)
C6—C5—C10	113.84 (17)	C20—C17—C13	114.68 (16)
C4—C5—C10	113.54 (18)	C16—C17—C13	104.10 (15)
C6—C5—H5	105.4	C20—C17—H17	108.3
C4—C5—H5	105.4	C16—C17—H17	108.3
C10—C5—H5	105.4	C13—C17—H17	108.3
C5—C6—C7	112.36 (18)	C13—C18—H18A	109.5
C5—C6—H6A	109.1	C13—C18—H18B	109.5
C7—C6—H6A	109.1	H18A—C18—H18B	109.5
C5—C6—H6B	109.1	C13—C18—H18C	109.5
C7—C6—H6B	109.1	H18A—C18—H18C	109.5
H6A—C6—H6B	107.9	H18B—C18—H18C	109.5
C8—C7—C6	111.84 (16)	C10—C19—H19A	109.5
C8—C7—H7A	109.2	C10—C19—H19B	109.5
C6—C7—H7A	109.2	H19A—C19—H19B	109.5
C8—C7—H7B	109.2	C10—C19—H19C	109.5
C6—C7—H7B	109.2	H19A—C19—H19C	109.5
H7A—C7—H7B	107.9	H19B—C19—H19C	109.5
C14—C8—C7	112.48 (16)	O1—C20—N1	122.7 (2)
C14—C8—C9	110.13 (16)	O1—C20—C17	122.2 (2)
C7—C8—C9	110.47 (17)	N1—C20—C17	115.0 (2)
C14—C8—H8	107.9	N1—C21—C23	106.2 (2)
C7—C8—H8	107.9	N1—C21—C24	110.37 (18)
C9—C8—H8	107.9	C23—C21—C24	109.7 (2)
C11—C9—C8	112.02 (16)	N1—C21—C22	109.20 (18)
C11—C9—C10	113.93 (16)	C23—C21—C22	111.0 (2)
C8—C9—C10	112.15 (15)	C24—C21—C22	110.2 (2)
C11—C9—H9	106.0	C21—C22—H22A	109.5
C8—C9—H9	106.0	C21—C22—H22B	109.5
C10—C9—H9	106.0	H22A—C22—H22B	109.5
C19—C10—C1	108.81 (17)	C21—C22—H22C	109.5
C19—C10—C5	112.08 (16)	H22A—C22—H22C	109.5
C1—C10—C5	106.56 (16)	H22B—C22—H22C	109.5
C19—C10—C9	110.26 (16)	C21—C23—H23A	109.5
C1—C10—C9	111.04 (15)	C21—C23—H23B	109.5
C5—C10—C9	108.05 (15)	H23A—C23—H23B	109.5
C9—C11—C12	114.43 (17)	C21—C23—H23C	109.5
C9—C11—H11A	108.7	H23A—C23—H23C	109.5
C12—C11—H11A	108.7	H23B—C23—H23C	109.5
C9—C11—H11B	108.7	C21—C24—H24A	109.5
C12—C11—H11B	108.7	C21—C24—H24B	109.5
H11A—C11—H11B	107.6	H24A—C24—H24B	109.5
C13—C12—C11	111.04 (16)	C21—C24—H24C	109.5
C13—C12—H12A	109.4	H24A—C24—H24C	109.5
C11—C12—H12A	109.4	H24B—C24—H24C	109.5
			
C10—C1—C2—C3	−54.5 (3)	C11—C12—C13—C18	−66.3 (2)
C1—C2—C3—C4	50.1 (3)	C11—C12—C13—C17	169.09 (17)
C2—C3—C4—C5	−50.5 (3)	C7—C8—C14—C15	−55.7 (3)
C3—C4—C5—C6	−173.8 (2)	C9—C8—C14—C15	−179.44 (17)
C3—C4—C5—C10	55.3 (3)	C7—C8—C14—C13	−179.00 (18)
C4—C5—C6—C7	175.0 (2)	C9—C8—C14—C13	57.3 (2)
C10—C5—C6—C7	−54.3 (2)	C12—C13—C14—C8	−61.5 (2)
C5—C6—C7—C8	53.1 (3)	C18—C13—C14—C8	60.3 (2)
C6—C7—C8—C14	−177.64 (18)	C17—C13—C14—C8	175.87 (17)
C6—C7—C8—C9	−54.1 (2)	C12—C13—C14—C15	167.69 (17)
C14—C8—C9—C11	−48.6 (2)	C18—C13—C14—C15	−70.46 (19)
C7—C8—C9—C11	−173.49 (17)	C17—C13—C14—C15	45.1 (2)
C14—C8—C9—C10	−178.19 (16)	C8—C14—C15—C16	−161.48 (18)
C7—C8—C9—C10	56.9 (2)	C13—C14—C15—C16	−33.8 (2)
C2—C1—C10—C19	−65.7 (2)	C14—C15—C16—C17	8.8 (2)
C2—C1—C10—C5	55.4 (2)	C15—C16—C17—C20	143.81 (18)
C2—C1—C10—C9	172.80 (17)	C15—C16—C17—C13	18.8 (2)
C6—C5—C10—C19	−67.1 (2)	C12—C13—C17—C20	81.4 (2)
C4—C5—C10—C19	63.0 (2)	C14—C13—C17—C20	−162.6 (2)
C6—C5—C10—C1	173.94 (18)	C18—C13—C17—C20	−44.0 (2)
C4—C5—C10—C1	−55.9 (2)	C12—C13—C17—C16	−154.74 (18)
C6—C5—C10—C9	54.5 (2)	C14—C13—C17—C16	−38.70 (19)
C4—C5—C10—C9	−175.31 (19)	C18—C13—C17—C16	79.89 (18)
C11—C9—C10—C19	−61.5 (2)	C21—N1—C20—O1	−4.2 (3)
C8—C9—C10—C19	67.1 (2)	C21—N1—C20—C17	172.60 (17)
C11—C9—C10—C1	59.2 (2)	C16—C17—C20—O1	−26.4 (3)
C8—C9—C10—C1	−172.21 (16)	C13—C17—C20—O1	92.6 (3)
C11—C9—C10—C5	175.74 (18)	C16—C17—C20—N1	156.77 (17)
C8—C9—C10—C5	−55.7 (2)	C13—C17—C20—N1	−84.2 (2)
C8—C9—C11—C12	48.2 (2)	C20—N1—C21—C23	−178.2 (2)
C10—C9—C11—C12	176.84 (18)	C20—N1—C21—C24	62.9 (3)
C9—C11—C12—C13	−53.2 (2)	C20—N1—C21—C22	−58.5 (3)
C11—C12—C13—C14	57.1 (2)		
